# A special role for the right posterior superior temporal sulcus during speech production

**DOI:** 10.1016/j.neuroimage.2019.116184

**Published:** 2019-12

**Authors:** Adam Kenji Yamamoto, Oiwi Parker Jones, Thomas M.H. Hope, Susan Prejawa, Marion Oberhuber, Philipp Ludersdorfer, Tarek A. Yousry, David W. Green, Cathy J. Price

**Affiliations:** a Neuroradiological Academic Unit, Department of Brain Repair and Rehabilitation, UCL Queen Square Institute of Neurology, University College London, Queen Square, London, United Kingdom; b Lysholm Department of Neuroradiology, National Hospital for Neurology and Neurosurgery, Queen Square, London, United Kingdom; c Wellcome Centre for Human Neuroimaging, UCL Queen Square Institute of Neurology, University College London, Queen Square, London, United Kingdom; d FMRIB Centre and Wolfson College, University of Oxford, Oxford, United Kingdom; e Collaborative Research Centre 1052 "Obesity Mechanisms", Faculty of Medicine, University of Leipzig, Leipzig, Germany; f Department of Neurology, Max Planck Institute for Human Cognitive and Brain Sciences, Leipzig, Germany; g Experimental Psychology, University College London, London, United Kingdom

**Keywords:** Speech production, Auditory feedback, Own speech, fMRI, RpSTS, right posterior superior temporal sulcus, LpSTS, left posterior superior temporal sulcus, RSTG, right superior temporal gyrus, LSTG, left superior temporal gyrus

## Abstract

This fMRI study of 24 healthy human participants investigated whether any part of the auditory cortex was more responsive to self-generated speech sounds compared to hearing another person speak. The results demonstrate a double dissociation in two different parts of the auditory cortex. In the right posterior superior temporal sulcus (RpSTS), activation was higher during speech production than listening to auditory stimuli, whereas in bilateral superior temporal gyri (STG), activation was higher for listening to auditory stimuli than during speech production. In the second part of the study, we investigated the function of the identified regions, by examining how activation changed across a range of listening and speech production tasks that systematically varied the demands on acoustic, semantic, phonological and orthographic processing. In RpSTS, activation during auditory conditions was higher in the absence of semantic cues, plausibly indicating increased attention to the spectral-temporal features of auditory inputs. In addition, RpSTS responded in the absence of any auditory inputs when participants were making one-back matching decisions on visually presented pseudowords. After analysing the influence of visual, phonological, semantic and orthographic processing, we propose that RpSTS (i) contributes to short term memory of speech sounds as well as (ii) spectral-temporal processing of auditory input and (iii) may play a role in integrating auditory expectations with auditory input. In contrast, activation in bilateral STG was sensitive to acoustic input and did not respond in the absence of auditory input. The special role of RpSTS during speech production therefore merits further investigation if we are to fully understand the neural mechanisms supporting speech production during speech acquisition, adult life, hearing loss and after brain injury.

## Highlights

• In right auditory cortex, a region is more sensitive to own than another’s speech.
• This region (RpSTS) responds to phonological input in the absence of auditory input.
• RpSTS may match auditory feedback with internal representations of speech sounds.

## 1 Introduction

This study investigates differences in the response of the auditory cortices to ones’s own speech compared to hearing another person speak when the conditions for auditory feedback are not experimentally perturbed. Previous functional neuroimaging investigations have demonstrated that the auditory cortices are activated during speech production (see Price, 2012 for review) but the response is significantly less than that observed when the same participants passively listen to recordings of their own speech (Christoffels et al., 2011, 2007; Greenlee et al., 2011; Kort et al., 2014). Auditory suppression, as it is usually termed, may serve to enhance the detection of external and informative auditory input from the environment, and appears to be related to articulatory activity in the motor cortex (Agnew et al., 2013; Parker Jones et al., 2013). Nevertheless, auditory processing of one’s own speech is needed to monitor and correct error-prone speech output. For example, when auditory feedback has been experimentally changed (perturbed) by shifting its frequency (Houde and Jordan, 1998; Niziolek and Guenther, 2013; Tourville et al., 2008), adjusting the syllable pitch (Behroozmand et al., 2015) or adding background noise (Zheng et al., 2010), functional imaging studies have shown that speech production activation increases in multiple bilateral superior temporal regions compared to when auditory feedback is not manipulated (Ventura et al., 2009). Here we examined whether any regions of the auditory cortices show enhanced activation to own speech compared to another’s speech, in the absence of experimental perturbation.

The possibility that different parts of auditory cortex are differentially sensitive to own and another’s speech, in unperturbed conditions, is consistent with animal vocalisation studies (Müller-Preuss and Ploog, 1981) and studies measuring electrocorticographic (ECoG) signals directly from the surface of the human auditory cortex (Flinker et al., 2010). In addition, two previous fMRI studies intimate this possibility but do not establish it. The first (Christoffels et al., 2007) noted increased activation during picture naming in the right posterior superior temporal sulcus (RpSTS) (at Montreal Neurological Institute (MNI) co-ordinates +49 -25 -2) for hearing one’s own speech compared to noise. A plot of the activation in the RpSTS (Fig. 3 in Christoffels et al., 2007) also indicates that RpSTS activation was higher for hearing one’s own speech while naming pictures than listening to recordings of own speech saying the same object names. This response in RpSTS contrasted with that in more dorsal bilateral superior temporal gyri (STG) where activation was higher (according to Fig. 3 in Christoffels et al., 2007) for listening to recordings of own speech in the absence of speech production than hearing own speech during speech production. However, the authors do not report the statistics for the direct comparison of hearing own speech during speech production compared to listening. Instead, the focus of the study was to highlight how the response to speech is reduced when participants are speaking.

The second study (Agnew et al., 2013) reported enhanced RpSTS activation (at +48 -31 ​+1), along with activation in the left posterior temporal lobe (at -42 -43 ​+1), for hearing own speech during reading aloud compared to listening to another’s speech while reading silently. This response in posterior temporal regions contrasted to that in the left superior temporal gyrus (at -60 -13 ​+4), where activation was higher for the reverse contrast (listening to another’s speech while reading silently compared to hearing own speech during reading aloud). The authors note the interesting dissociation between anterior and posterior temporal regions but did not discuss the posterior regions because their study focused on the suppression of the anterior temporal activation in the presence of articulatory activity. In addition, we note that the effect of own compared to another’s speech in the right posterior superior temporal sulcus would not be significant after correction for multiple comparisons.

Based on the studies reported by Christoffels et al. (2007) and Agnew et al. (2013), our hypothesis in the current study is that RpSTS, and/or other auditory processing regions, will be more activated by own speech during speech production than another’s speech that is being listened to. The alternative hypothesis, however, is that the increased activation in RpSTS during speech production compared to listening (Christoffels et al., 2007; Agnew et al., 2013) reflected higher attention to auditory inputs during speech production than during passive listening in the absence of an attention demanding task. In our study, we therefore used an active listening task that required participants to attend to auditory stimuli and hold them in memory during one-back matching.

In the second part of our study, we investigated the response properties of the auditory processing regions (e.g. RpSTS) that were more activated by own speech than another’s speech so that we can better understand the type of speech production processing. For example, are they sensitive to the duration or type of acoustic input (e.g. speech stimuli versus non-speech stimuli) and do they also respond to inner speech processing (phonology) in the absence of auditory input or output.

To investigate the response properties of the auditory areas that were more activated by own than another’s speech, our experimental design systematically manipulated the demands on sensory input, semantic content, sublexical phonological cues and task. Using this design we identified: (i) a set of auditory processing regions that were more activated for auditory than visual conditions, after controlling for task, semantics and phonology, (ii) which parts of these auditory processing regions were more activated for speech production (own speech) compared to hearing another’s speech, (iii) whether these regions were sensitive to the demands on semantic, phonological, or orthographic processing and (iv) whether these regions responded in the absence of auditory input – as would be expected if they are involved in auditory expectations that are generated during articulatory activity (Agnew et al., 2013).

## 2 Materials and methods

### 2.1 Participants

Twenty four, healthy, right handed English speakers (12 female, 12 male) participated in the study. Their mean age was 31.4 years, standard deviation (SD)  =  5.9 years; range  =  20–45). Handedness was assessed with the Edinburgh Handedness Inventory (Oldfield, 1971). All subjects gave written informed consent prior to scanning with ethical approval from the London Queen Square Research Ethics Committee.

### 2.2 Experimental design

The fMRI experiment comprised a 2 x 2 x 2 x 2 factorial design allowing us to dissociate brain activity related to experimental task (speech production versus one-back matching); modality (auditory compared to visual stimuli); semantic content (words and meaningful pictures or sounds versus pseudowords and meaningless pictures or sounds) and sublexical phonological cues that facilitate the perception or retrieval of phonological representations (e.g. English words and pseudowords compared to pictures and nonverbal sounds). Data from this paradigm have previously been reported in Hope et al. (2014) to dissect the functional anatomy of auditory repetition.

The speech production tasks with auditory stimuli were: auditory repetition of heard object names (with sublexical phonological cues and semantic content), auditory repetition of pseudowords (with sublexical phonological cues without semantic content), naming aloud objects from their sounds (with semantic content without sublexical phonological cues) and naming aloud the gender of the voice heard producing meaningless humming (without semantic or sublexical phonological cues). The speech production tasks with visual stimuli were: reading aloud object names (with sublexical phonological cues and semantic content), reading aloud pseudowords (with sublexical phonological cues, without semantic content), naming objects from pictures (with semantic content, without sublexical phonological cues), and naming the colour of meaningless non-objects (without semantic content or sublexical phonological cues). The participants were presented with exactly the same stimuli (both auditory and visual) while performing a silent one-back matching task (in other words, each participant saw the same stimuli in the speech production and one-back matching conditions), see Sections 2.4, 2.5 for more details.

None of the analyses or conclusions reported in the current study have been included in previous studies.

### 2.3 Stimulus selection/creation

We selected 128 names of familiar objects and animals. The written versions of the names had 3 to 12 letters (mean  =  5 letters, SD = 1.8), corresponding to one to four syllables (mean  =  1.59, SD = 0.73). The auditory versions of these names were recorded by a native, male, English speaker (with a Southern British accent approximating Received Pronunciation) while reading aloud the written versions at the same rate that they were presented in the experiment (see below). The duration of these auditory stimuli ranged from in 0.48–0.95  seconds (s) (mean duration = 0.64 s, SD = 0.1).

**Fig. 1:**
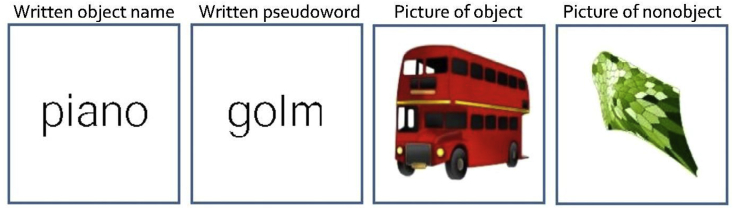
Examples of the visual stimuli.

The pictures of the 128 objects were drawn for the purposes of this experiment by a professional artist (Eldad Druks). They were drawn as realistically as possible in colour with key features outlined in black to ensure they were easily recognisable in the scanner (see Fig. 1). This was confirmed by the high naming accuracy.

The sounds of the objects were taken from the NESSTI sound library (Hocking et al., 2013) but only 32 of our 128 objects had sounds that were unambiguously related to one object/animal. For example, while it is easy to recognise that the source of a dog barking is a dog, it is not easy to individually recognise most object/animal sounds without other clues (e.g. kangaroo, panda bear and table sounds). The effect of this stimulus limitation is discussed in Sections 2.4, 2.5 but we also note here that there was no possible impact of stimulus confounds on our results because none of our effects were specific to the sound condition. The mean duration of these 32 sounds (1.47 s, SD  =  0.13) was significantly longer (t_126_ = 37.8, p < 0.001) than that of the auditory object names (mean duration = 0.64 s, SD  = 0.1) with this difference taken into consideration when interpreting the results.

Pseudowords were created using a nonword generator (Duyck et al., 2004). To ensure that the pseudoword stimuli were balanced with the word stimuli, we generated 128 written pseudowords that were matched to the 128 objects names for bigram frequency, number of orthographic neighbours and word length. Auditory pseudowords were recorded in the same way as the words by the same speaker.

The visual non-semantic, non-phonological stimuli were “coloured non-objects” (see Fig. 1) created from the object pictures by scrambling the global and local features to render them unrecognisable and then manually editing the images to accentuate one of eight colours (brown, blue, orange, red, yellow, pink, purple and green). The colours were not uniform in either the object or non-object conditions (see Fig. 1). Pilot studies ensured that the fMRI participants would agree on the colour of each stimulus. The visual form and colour shade changed on each trial, but each of the colour names appeared four times (32 stimuli in total) per scan run.

The auditory non-semantic, non-phonological stimuli were created by male or female voices humming for approximately 1 s (mean length  =  1.04 s, SD  =  0.43) with no phonological or semantic content. Half the hums were matched in length to the words (mean duration  =  0.64 s) and the other half were matched in length to the object sounds (mean duration  =  1.47 s). This allowed us to investigate the effect of acoustic duration on activation in our regions of interest.

### 2.4 Stimulus assignment to different conditions

There were four different types of object stimuli used in this experiment: (i) pictures of objects/animals, (ii) sounds of objects/animals, (iii) visually presented (written) object/animal names, and (iv) auditory presented (heard) object/animal names. To assign stimuli to different conditions, we divided the 128 object names into four sets of 32 (A, B, C, D). Sets A-C were rotated across pictures of objects, visual object names and auditory object names, in different participants so that (i) all items were novel on the first presentation of each stimulus type and (ii) the semantic and phonological content of these three conditions was matched across subjects.

Set D included the sounds of 32 objects that were always used during the object sound conditions and never used in any other condition. The semantic content of the auditory object stimuli was therefore not matched to the other object conditions (visual objects, auditory words or visually presented words). The auditory object sounds were also longer than auditory words because otherwise they were not recognisable. To facilitate object recognition from sounds, and ensure high accuracy for auditory sound naming, all participants were familiarised with the sounds prior to scanning whereas they were not familiarised with any of the other stimuli. These inter-condition differences do not confound any of the results we report. For example, with respect to the main effect of task, each participant saw exactly the same stimuli in the speech production and one-back matching conditions. Task differences were therefore independent of stimulus content, and were fully counterbalanced across 24 subjects (see Section 2.5 for counterbalancing). With respect to the main effect of sensory input (auditory  >  visual), we looked for differences that were consistent across condition, i.e. common for (i) repeating words  >  reading words, (ii) repeating pseudowords  >  reading pseudowords, (iii) naming objects from sounds  >  pictures and (iv) naming gender  >  colour. As contrasts (i) and (ii) presented exactly the same words in the auditory and visual conditions, they were matched for phonological and semantic content. Therefore, any common differences across contrasts (i), (ii), (iii) and (iv) could not be attributed to stimulus confounds/object content. To the contrary, common effects that generalise across stimuli with different semantic and phonological content, ensure the generalisability of our main effects of interest.

Auditory pseudowords (with phonological but not semantic content) were matched to the set of objects that were presented as pictures (with semantic but not phonological content). Likewise, written pseudowords were matched to the set of objects presented as sounds (i.e. Set D). The goal here was to match word length, bigram frequency and number of orthographic neighbours across (i) the phonological only and semantic only conditions and (ii) the visual and auditory conditions. Indeed, the final set of results did not reveal any results that could be influenced by any remaining stimulus confounds because the main effects of interest in our area of interest did not interact with stimulus modality, semantics or phonology.

### 2.5 Counterbalancing

Half the participants (12/24) performed all 8 speech production tasks first and then the 8 one-back matching tasks (on exactly the same set of stimuli as seen/heard in the speech production conditions). The other half (12/24) performed the one-back matching first and then the speech production tasks (on exactly the same stimuli). Within each task, the order of conditions was fully counterbalanced across 24 participants.

We split each set of 32 items into four blocks of eight stimuli with one of the eight stimuli repeated in each block to make a total of nine stimuli per block (eight novel, one repeat). The stimulus repeat only needed to be detected and responded to (with a finger press) in the one-back matching tasks but was also present in the speech production conditions in order to keep the stimuli constant across tasks and participants.

### 2.6 Procedure

Prior to scanning, we trained each participant on all tasks using a separate set of training stimuli except for the environmental sounds which remained the same. All speaking tasks required the participant to produce a single spoken response after each stimulus presentation by saying aloud the object name, pseudoword, colour name and either ‘male or female’ in response to the hum. Pilot testing indicated that participants could hear their own speech when wearing earphones and this was consistently accompanied by highly significant activity in the auditory cortices relative to scanner noise alone. We are not concerned here as to whether this was driven by bone conduction or air conduction.

For the one-back matching task, participants placed two fingers of the same hand (12 participants used the right hand, and the other 12 used the left) over an fMRI compatible button box to indicate whether the stimulus was the same as the one preceding it (left button for ‘same’, right button for ‘different’). There was no overt speech production involved in any one-back matching condition. During both visual and auditory conditions, participants were instructed to respond as fast as possible, keeping their body and head as still as possible and their eyes open and fixated on a cross in the middle of the display screen. An eye tracker was used to constantly monitor the participants’ eyes. This allowed us to confirm that all participants had their eyes open and paid constant attention throughout the experiment.

Each of the 16 tasks was presented in a separate scan run, all of which were identical in structure. The script was written with COGENT (http://www.vislab.ucl.ac.uk/cogent.php) and run in Matlab 2010a (Mathsworks, Sherbon, MA, USA, RRID:SCR_001622). Scanning started with the instructions ‘Get Ready’ written on the in-scanner screen while five dummy scans were acquired (15.4 s in total). This was followed by a written instruction (e.g. ‘Repeat’), lasting 3.08 s, which indicated the forthcoming start of a new block and reminded participants of the task that needed to be performed. Each block of stimuli presented nine stimuli with an inter-stimulus interval of 2.52 s (total block length  =  22.68 s) and was followed by 16 s fixation. The instructions, stimuli and fixation was repeated four times resulting in just over 3  min of scanning per run.

Each visual stimulus was displayed for 1.5 s, followed by 1.02 s fixation until the next stimulus. The rate of stimulus presentation was the same for auditory and visual stimuli (always 2.52 s), however, the stimulus:fixation ratio varied for each stimulus. Means (and standard deviations) for the duration of auditory stimuli were 0.64 (0.10) for auditory words, 0.68 (0.12) for auditory pseudowords, 1.47 (0.12) for object sounds and 1.04 (0.43) for humming sounds.

The pictures subtended an angle of 7.4**°** (10  cm on screen, 78  cm viewing distance) with a pixel size of 350  ×  350, and a screen resolution of 1024  ×  768. The visual angle for the written words ranged from 1.47 to 4.41**°**, with the majority of words (with five letters) extending 1.84–2.2**°**. Auditory stimuli were presented via MRI compatible headphones (MR Confon, Magdeburg, Germany), which filtered ambient in-scanner noise. Volume levels were adjusted for each participant before scanning. Spoken responses were recorded via a noise-cancelling MRI microphone (FOMRI IIITM Optoacoustics, Or-Yehuda, Israel), and transcribed manually for off-line analysis.

In-scanner behaviour was measured for each of the 16 conditions. Correct responses were those that matched the target without delay or self-correction. All other responses were categorised as incorrect. For one-back matching, accuracy and response times (from stimulus onset to button press) were computed automatically, according to the button pressed in response to each trial. For speech production, spoken responses were recorded via a microphone and monitored by the experimenter who either (i) ticked a check list to confirm that the expected response had been made or (ii) recorded an alternative (or null) response. For some stimuli, more than one response was considered corrected. For example, a picture of a mug could be named “cup” or “mug”. The same criteria were used for all participants. Response times for speech production were analysed off-line. Unfortunately, however, it was not possible to accurately record speech onset times and therefore these data are not reported in the current study. The accuracy of responses was used in the fMRI analysis to disambiguate activation for correct trials (of interest) from activation related to incorrect trials (not of interest).

Response times for correct one-back matching trials were analysed in SPSS (IBM SPSS 22, NY, USA). To test for main effects and interactions we conducted a repeated measures 2  ×  2  ×  2 ANOVA. Factor 1 stimulus modality (visual vs. auditory), factor 2 was semantic content (words and objects versus pseudowords and baseline) and factor 3 was sublexical phonological content (words and pseudowords more than objects and baseline).

### 2.7 Data acquisition

Functional and anatomical data were collected on a 3T scanner (Trio, Siemens, Erlangen, Germany) using a 12-channel head coil. To minimise movement during acquisition, a careful head fixation procedure was used when positioning each participant’s head. This ensured that none of the speech sessions were excluded after checking the realignment parameters. Functional images consisted of a gradient-echo planar imaging (EPI) sequence and 3  ×  3 mm^2^ in-plane resolution (TR/TE/flip angle  =  3080 milliseconds (ms)/30  ms/90**°**), field of view (EFOV) = 192  mm, matrix size  =  64  ×  64, 44 slices, slice thickness  =  2  mm, interslice gap  =  1  mm, 62 image volumes per time series, including five “dummies” to allow for T1 equilibration effects. The TR was chosen to maximize whole brain coverage (44 slices) and to ensure that slice acquisition onset was offset-asynchronised with stimulus onset, which allowed for distributed sampling of slice acquisition across the study (Veltman et al., 2002). For anatomical reference, a high-resolution T1 weighted (w) structural image was acquired after completing the tasks using a three-dimensional Modified Driven Equilibrium Fourier transform (MDEFT) sequence (TR/TE/TI  =  7.92  ms/2.48  ms/910  ms), flip angle  =  16**°**, 176 slices, voxel size  =  1 × 1 × 1 mm^3^). The total scanning time was approximately 1  h and 20  min per participant, including set-up and the acquisition of the anatomical scan.

### 2.8 fMRI data preprocessing

Data preprocessing and statistical analysis were performed in SPM12 (Wellcome Trust Centre for Neuroimaging, London, UK), running on MATLAB 2012a. Functional volumes were spatially realigned to the first EPI volume and unwarped to compensate for non-linear distortions caused by head movement or magnetic field inhomogeneity. The unwarping procedure was used in preference to including the realignment parameters as linear regressors in the first-level analysis because unwarping accounts for non-linear movement effects by modelling the interaction between movement and any inhomogeneity in the T2* signal. After realignment and unwarping, the realignment parameters were checked to ensure that participants moved less than one voxel (3 ​mm) within each scanning run.

The anatomical T1w image was co-registered to the mean EPI image generated during the realignment step and then spatially normalised to the MNI space using the new unified normalisation-segmentation tool of SPM12. To spatially normalise all EPI scans to MNI space, the deformation field parameters that were obtained during the normalisation of the anatomical T1w image were applied. The original resolution of the different images was maintained during normalisation (voxel size 1  ×  1  ×  1  mm^3^ for structural T1w and 3  ×  3  ×  3  mm^3^ for EPI images). After the normalisation procedure, functional images were spatially smoothed with a 6  mm full-width-half-maximum isotropic Gaussian Kernel to compensate for residual anatomical variability and to permit application of Gaussian random-field theory for statistical inference (Friston et al., 1995).

### 2.9 First level statistical analyses

Each preprocessed functional volume was entered into a subject specific fixed effect analysis using the general linear model. Stimulus onset times were modelled as single events with two regressors per run, one modelling the instructions and one modelling all stimuli of interest (including repeated and unrepeated items). Stimulus functions were convolved with a canonical haemodynamic response function and high pass filtered with a cut-off period of 128  s.

For each scanning session/run (that alternated one condition of interest with fixation), we generated a single contrast that compared (A) activation in response to the stimuli and task of interest to (B) baseline activation during resting with fixation. This resulted in 16 different contrasts (one per condition) for each participant. Each contrast for each individual was inspected to ensure that there were no visible artefacts (e.g. edge effects, activation in ventricles) that might have been caused by within-scan head movements.

### 2.10 Second level statistical analyses

At the second level, the 16 contrasts for each participant were entered into a within-subjects one-way ANOVA in SPM12. Main effects and interactions were computed at the contrast level. First, we created regions of interest in the auditory cortices that were more activated for the main effect of auditory compared to visual stimuli (see Table 1, contrast a). Second, within these regions, we identified which parts were also activated by the main effect of speech production (Table 1, contrast b). If we had not limited our analysis of speech production to auditory processing regions, greater activation for speech production may have been a consequence of motor output rather than auditory processing of the spoken response. Third, within the regions commonly activated by the main effect of auditory input and the main effect of speech production, we identified which parts were more activated by the main effect of speech production than the main effect of auditory input (Table 1 contrast c) and which parts were more activated by the main effect of auditory input than the main effect of speech production (i.e. the reverse of contrast c, c2). Fourth, within the regions that were more or less sensitive to speech production, we report the main effects of semantics (contrast d), phonology (contrast e) and the interaction between phonological content and sensory modality (contrast f, orthographic to phonological processing occurring for phonological input in the visual not auditory modality. We also test for the reverse of contrasts d and e (contrast d2, contrast e2). Finally, we test whether auditory areas that respond during speech production are also activated in the absence of auditory input (i.e. during one-back matching of visual stimuli).

**Table 1:** Experimental conditions and statistical contrasts. SP is speech production, OBM is one-back matching. Contrast (a) is the main effect of Auditory > Visual conditions. The reverse of this contrast is the main effect of visual input which was not of interest. Contrast (b) is the main effect of speech production compared to one-back matching on exactly the same stimuli. The reverse of this contrast is the main effect of one-back matching which was not of interest. Contrast (c) identified areas where the main effect of speech production (contrast b) was greater than the main effect of auditory input (contrast a) (contrast c = b - a). This is only reported in auditory processing areas (i.e. significant in contrast (a) that also showed an effect of speech production (i.e. significant in contrast (b)), so controlling for all other variables. (c2) is the reverse of contrast (c) and identified areas where the main effect of auditory input (contrast a) was greater than the main effect of speech production (contrast b). Contrast (d) identified the main effect of semantic content (Sem) by comparing pictures, sounds and names of objects to the other conditions. We also tested the reverse of this contrast, (d2). Contrast (e) identified the main effect of sublexical phonological cues to speech production (Phon) by comparing words and pseudowords to all other conditions. We also tested the reverse of this contrast, (e2). Contrast (f) identified whether the effect of words/pseudowords (phonological inputs) was greater in the written domain (orthographic) compared with the auditory domain. The reverse of this contrast (phonological content in the auditory > visual domain) tests for activation related to auditory speech sounds.

Conditions			Statistical contrasts								
			a	b	c	c2	d	d2	e	e2	f
Task	Input	Stimulus	Aud	SP	SP-Aud		Sem		Phon		Orth
SP	Visual	Pictures of objects	−1	1	2	−2	1	−1	−1	1	−1
		Words	−1	1	2	−2	1	−1	1	−1	1
		Pseudowords	−1	1	2	−2	−1	1	1	−1	1
		Coloured non-objects	−1	1	2	−2	−1	1	−1	1	−1
	Auditory	Sounds of objects	1	1	0	0	1	−1	−1	1	1
		Words	1	1	0	0	1	−1	1	−1	−1
		Pseudowords	1	1	0	0	−1	1	1	−1	−1
		Baseline (Humming)	1	1	0	0	−1	1	−1	1	1
OBM	Visual	Pictures of objects	−1	−1	0	0	1	−1	−1	1	−1
		Words	−1	−1	0	0	1	−1	1	−1	1
		Pseudowords	−1	−1	0	0	−1	1	1	−1	1
		Coloured non-objects	−1	−1	0	0	−1	1	−1	1	−1
	Auditory	Sounds of objects	1	−1	−2	2	1	−1	−1	1	1
		Words	1	−1	−2	2	1	−1	1	−1	−1
		Pseudowords	1	−1	−2	2	−1	1	1	−1	−1
		Baseline (Humming)	1	−1	−2	2	−1	1	−1	1	1

#### 2.10.1 Statistical thresholds

The statistical threshold for the main effects of auditory input and speech production (contrasts (a) and (b) in Table 1) was set at p  <  0.05, after family wise error correction for multiple comparisons in each voxel across the whole brain. For the remaining effects, the statistical contrasts were set at p  <  0.05 after family wise error correction for multiple comparisons at each voxel within our regions of interest (ROI). The ROI were spheres (6  mm radius) centred on the MNI co-ordinates reported for own and another’s speech in Agnew et al. (2013). For own more than another’s speech, these were: (+48 -31 ​+1) in the right RpSTS and (-42 -43 ​+1) in the left posterior temporal lobe. For another’s more than own speech, the co-ordinates were (-60 -13 ​+4) in the left superior temporal gyrus (LSTG). We also investigated the response in the hemispheric homologue of all these regions, i.e. (-48 -31 ​+1) in the left posterior superior temporal sulcus (LpSTS), (+42 -43 ​+1] in the right posterior temporal lobe and (+60 -13 ​+4) in the right superior temporal gyrus (RSTG).

#### 2.10.2 Post hoc analysis of hemispheric differences

To statistically confirm that pSTS responses for speech production were higher in the right than left hemisphere, and that this hemisphere effect was significantly different in pSTS than STG, we extracted the data from RpSTS, LpSTS, RSTG and LSTG and analysed how hemisphere and region interacted with our four variables (task, modality, semantics and phonology). The resulting 2 x 2 x 2 x 2 x 2 x 2 analysis was conducted in SPSS using a repeated measures ANOVA and a statistical threshold of p  <  0.05 (2-tailed). Data were extracted using the principal eigenvariate function in SPM from the voxel with the peak response to speech production  >  auditory input (contrast c) for RpSTS and LpSTS and the reverse contrast (auditory input  >  speech production, contrast c2) for RSTG and LSTG. We chose peak voxels from the results of these contrasts rather than the co-ordinates from Agnew et al. (2013), to avoid over-estimating right-laterality in pSTS, given that, as expected, none of the voxels in the homologue of RpSTS (i.e. LpSTS) reached significance in the Agnew et al. region of interest.

## 3 Results

### 3.1 Behavioural results

In scanner accuracy was high for all conditions (Table 2a). Response times (RTs) during one-back matching were available for all conditions in 21 participants. Response times for the other three participants were excluded from the RT analysis because of technical failure with the response pad on one or two of the 16 conditions. The mean RTs per condition are reported in Table 2a. Statistical analyses (see Table 2b for details) indicated that RTs were significantly faster for (i) visual stimuli (that are fully delivered at trial onset) than auditory stimuli (that are delivered sequentially); (ii) phonological stimuli (words and pseudowords) than non-phonological stimuli (pictures of objects and baseline conditions) and this phonological effect was stronger in the auditory than visual modality; (iii) semantic stimuli than non-semantic stimuli (words faster than pseudowords; and objects faster than baselines) and this effect was greatest for phonological stimuli (words faster than pseudowords) in the auditory than visual modality but for non-phonological stimuli (objects faster than baseline) in the visual than auditory modality (see Table 2b for effect sizes and statistical details).

**Table 2a:** In scanner behavioural results.

Modality	Stimulus	Duration	RT	Accuracy	
			OBM	OBM	SP
Visual	Objects (O)	1500	683 (115.7)	99.7 (0.8)	96.0 (4.6)
	Words (W)	1500	655 (113.1)	97.7 (5.8)	99.6 (1.3)
	Pseudowords (Ps)	1500	648 (88.4)	98.6 (4.3)	85.8 (15.1)
	Colours (C)	1500	762 (111.0)	95.6 (2.9)	99.0 (1.9)
Auditory	Objects (O)	1470 (120)	1111 (330.6)	96.7 (5.9)	91.8 (7.6)
	Words (W)	640 (100)	880 (113.7)	99.1 (3.0)	99.5 (1.1)
	Pseudowords (Ps)	680 (120)	959 (136.1)	99.1 (1.6)	88.3 (8.7)
	Humming (H)	1040 (430)	1125 (226.4)	88.8 (9.7)	99.1 (2.1)

SP is speech production, OBM is one-back matching. Duration refers to length of stimulus presentation in ms (standard deviation). RT refers to response times in ms (standard deviation) that were only available for one-back matching. Accuracy is the mean percentage of correct responses with standard deviation.

**Table 2b:** Results of repeated measures ANOVA on OBM response times.

Effect	F	Df	P value	Post hoc analysis (see Table 2a)
Modality (Mod)	146.6	1,20	0.000	Faster for Visual (vis) than Auditory (Aud)
Phonology (Phon)	35.2	1,20	0.000	Faster for W & Ps than Obj & C/H
Semantics (Sem)	4.9	1,20	0.038	Faster for W than Ps, & for Obj than C/H
Mod x Phon	8.5	1,20	0.009	Phon effect is bigger for Aud than Vis stimuli
Mod x Phon x Sem	7.6	1,20	0.012	Sem effect is bigger for Aud phon (W < Ps); and Sem effect is bigger for Vis non-phon (O < C)
Mod x Sem	0.115	1,20	0.738	Not significant
Phon x Sem	0.053	1,20	0.821	Not significant

‘x’ denotes the testing of an interaction.

Response times during one-back matching were longer for auditory stimuli with longer durations (sounds and vocal humming) than those with shorter durations (words and pseudowords), see Table 2a. This can be explained because the time to present the stimuli was longer for object sounds than auditory speech.

### 3.2 fMRI results

#### 3.2.1 The main effect of speech production in auditory processing regions

**Fig. 2:**
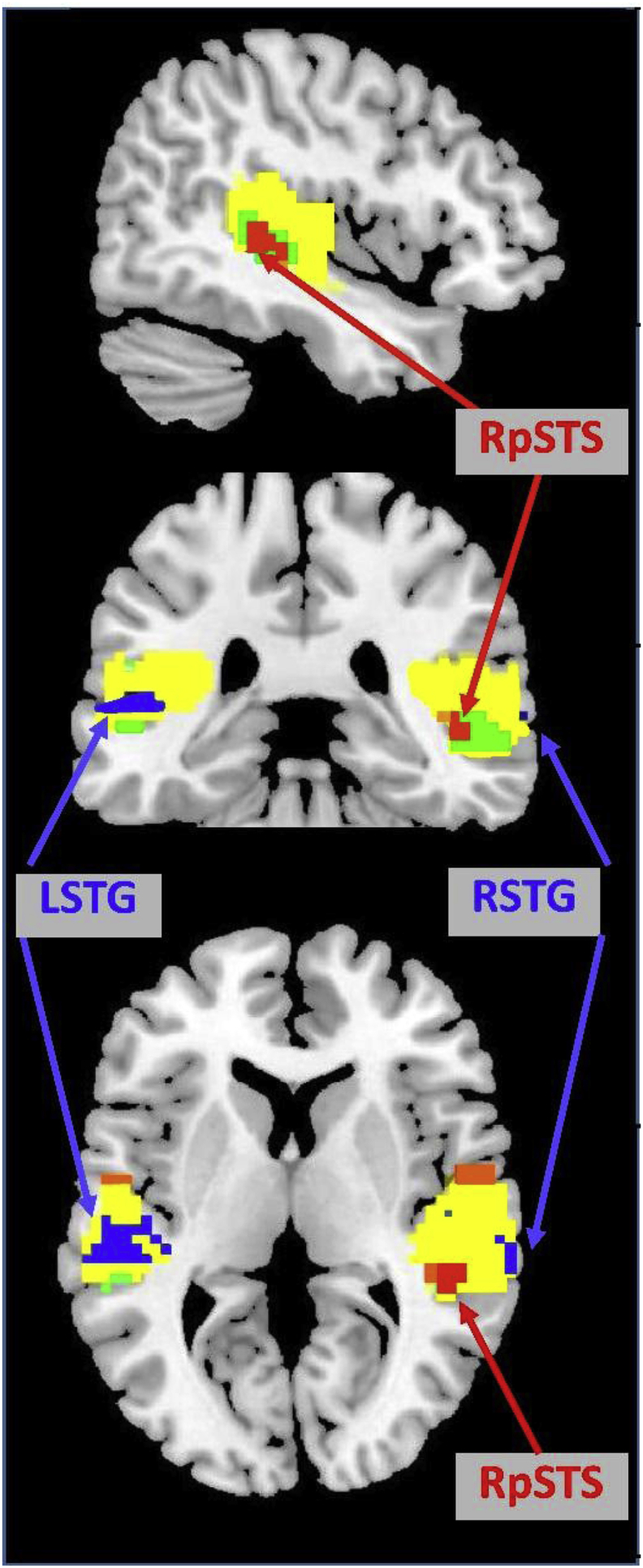
Superior temporal lobe activation for processing own and another’s speech. Sagittal (top), coronal (middle) and axial (bottom) brain slices (at MNI co-ordinates: +45 -33 ​+6) showing regions of interest in the auditory cortices. All coloured regions (yellow, red, orange, blue and green) were activated by main effect of auditory input and main effect of speech production (both at p ​< ​0.05 corrected for multiple comparisons across the whole brain). Blue areas show the LSTG and RSTG regions that were more activated by hearing another’s speech than own speech (contrast c2 in ​Table 1). The red RpSTS region was more activated by (i) speech production than listening to another’s speech (contrast c in ​Table 1) and (ii) one-back matching on written pseudowords compared to rest. The orange bilateral regions bordering the ventral surface of the premotor cortex were also more activated for speech production than listening but are not discussed because they were not in regions of interest and activation was explained by motor activity during speech production. Green regions were activated by one-back matching on written pseudowords compared to rest but are not of interest because they were not more activated by speech production compared to listening to another’s speech. Yellow regions show the remaining auditory input areas activated for the main effects of both auditory input and speech production. Blue, red/orange and green areas include all voxels that surpassed a threshold of p <  0.01 uncorrected to show the full extent of activation around peaks that survived significance after correction for multiple comparisons in regions of interest.

Significant activation for the main effects of (i) auditory compared to visual stimuli (contrast a in Table 1) and (ii) speech production compared to one-back matching (contrast b in Table 1) was observed in RpSTS, LpSTS, RSTG, LSTG, see Table 3 for Z scores and p values. This combination of effects suggests that all four regions of interest were involved in auditory processing of the participants own speech, see Fig. 2 for the extent of these effects across the auditory cortices.

**Table 3:** fMRI activation results in regions of interest. The contrast labels (a to f) in the first column correspond to those detailed in ​Table 1. ‘x’ ​denotes the testing of an interaction. The regions of interest are centred on the areas reported in ​Agnew et al. (2013) ​for own versus another’s speech in right pSTS (RpSTS) (x ​= ​+48, y ​= ​-31, z ​= ​+1) and other versus own speech in left STG (LSTG) (-60 -13 ​+ ​4). Effects are also reported in the homologues of these regions: left pSTS (LpSTS) (-48 -31 ​+ ​1) and right STG (RSTG) (+60 -13 ​+ ​4). P-values are corrected for multiple comparisons across the whole brain, unless appended with a u (i.e. p ​< ​0.001u) which indicates uncorrected thresholds or * which indicates a small volume correction for multiple comparisons in the regions of interest and for the effects of interest (i.e. RpSTS for speech production ​> ​auditory processing and LSTG for auditory processing ​> ​speech production). ns = not significant.

Contrast		RpSTS		LpSTS		RSTG		LSTG	
		Z	P	Z	P	Z	P	Z	P
(a)	Auditory > Visual	10.3	*<0.001*	13.5	*<0.001*	21.8	*<0.001*	16.0	*<0.001*
(b)	SP > OBM	11.2	*<0.001*	5.3	*<0.003*	10.3	*<0.001*	7.8	*<0.001*
(c)	SP > Auditory	3.6	0.004*	∼	ns	∼	ns	∼	ns
(c2)	Auditory > SP	∼	ns	∼	ns	6.0	*<0.001*	5.3	*<0.001*
(d2)	Non-Sem > Sem	*4.4*	*<0.001u*	∼	ns	∼	ns	∼	ns
(d2) x (a)	Non-sem > Sem for Auditory > Visual	*3.6*	*<0.001u*	∼	ns	∼	ns	∼	ns
(e2)	Non-phon > Phon	*∼*	*ns*	∼	ns	4.0	*<0.001u*	∼	ns
(e2) x (a)	Non-phon > Phon for Auditory > Visual	*∼*	*ns*	6.3	*<0.001*	7.2	*<0.05*	4.8	*<0.001u*
(f)	Phon from orthography > no orthography	*3.5*	*<0.001u*	∼	ns	∼	ns	∼	ns

**Fig. 3:**
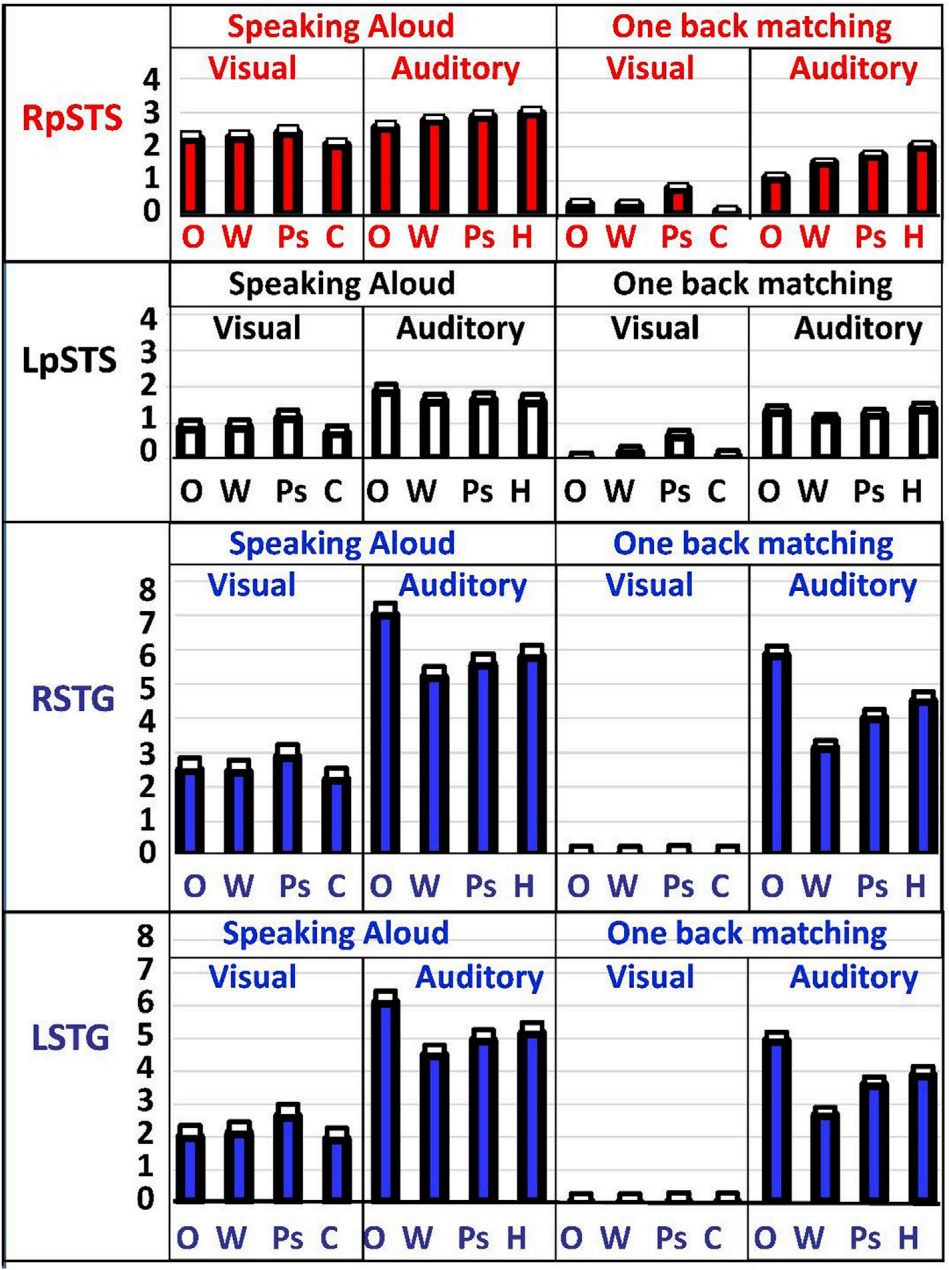
Condition specific responses in left and right pSTS and STG. Activation for each of the four regions in each of the 16 conditions. Going from left to right, conditions 1–8 ​= ​speech production, conditions 9–16 ​= ​one-back matching. Conditions 1–4 and 9–12 ​= ​visual stimuli. Conditions 5–8 and 13–16 ​= ​auditory stimuli. O ​= ​object naming from pictures (visual) or sounds (auditory), W ​= ​words, Ps ​= ​pseudowords, C ​= ​coloured non-objects, H ​= ​male and female humming. Activation is plotted at the voxels, within our regions of interest, showing the peak effect of speech production more than auditory input (contrast c) for RpSTS and LpSTS and the peak effect of the reverse contrast for RSTG and LSTG. These co-ordinates were: (+45 -33 ​+3), (-48 -31 ​+1), (+60 -15 ​+3) and (-57 -15 0). The plots are colour coded to help link the plot to the regions shown in ​Fig. 2. The plot showing LpSTS is not coloured because there was no significant effect of own or another’s speech in this region. The peak is included for comparison with RpSTS. Standard errors are marked in white boxes above the mean response for each condition.

In the left temporal region that Agnew et al. (2013) reported for reading aloud compared to reading silently while listening to another’s speech (at -42 -43 ​+1), we did not find significant activation for the main effect of auditory processing or the main effect of speech production. Therefore we do not report any further details about this area.

#### 3.2.2 Auditory areas where the effect of speech production is higher than the effect of auditory processing

Within the auditory processing regions that were commonly activated by the main effects of auditory than visual processing and speech production than one-back matching, RpSTS was, as predicted, more activated for speech production than auditory processing (contrast c, Table 1 and Fig. 2). This effect was not observed in RSTG, LpSTS or LSTG (see Table 3 for statistical details). Within the RpSTS region of interest, the peak voxel was located at (x ​= ​+45, y ​= ​-33, z ​= ​+3). No corresponding effect was identified in the LpSTS.

Higher RpSTS activation for speech production than auditory processing was observed even when the stimuli heard during speech production had the same semantic and phonological content as the stimuli heard during one-back matching (i.e. the set of words and pseudowords that were read aloud in the speech production conditions were the same as the set of words and pseudowords that were heard in another’s voice during one-back matching), see Fig. 3.

#### 3.2.3 Auditory areas where the effect of auditory processing is higher than the effect of speech production

More activation for the main effect of auditory input than the main effect of speech production (the reverse of contrast c, c2, in Table 1), was observed in both the left and right superior temporal regions of interest (LSTG and RSTG), see Table 3 and blue areas in Fig. 2. This effect was observed even when the speech heard during speech production had the same semantic and phonological content as the stimuli heard during one-back matching (i.e. the word and pseudoword conditions), see Fig. 3.

#### 3.2.4 Hemispheric dominance in pSTS

The SPSS analysis tested whether speech production activation was stronger in right than left pSTS and was based on data extracted from the co-ordinates showing the highest activation differences between speech production and auditory input in our regions of interest. These were identified for: RpSTS at: (+45 -33 ​+3), left pSTS at (-48 -31 ​+1), RSTG at (+60 -15 ​+3) and LSTG at (-57 -15 0).

**Fig. 4:**
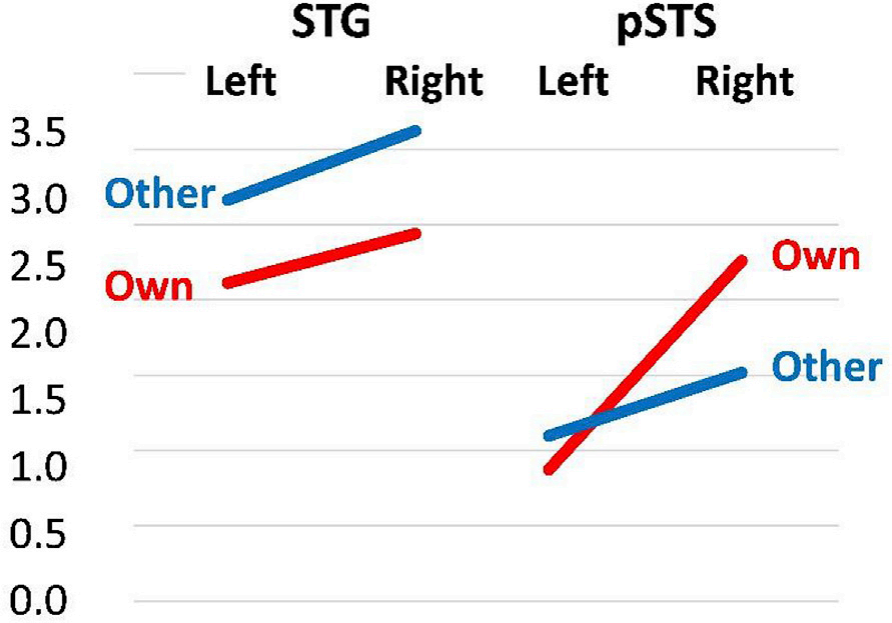
Contrasting effects in bilateral STG and RpSTS. This figure illustrates the task by hemisphere interaction for the word conditions only. Other =  other speech when listening to words and performing the one-back matching task. Own  =  own speech production when the same words were read aloud. These two tasks were selected because (i) they segregate other speech (listening) from own speech (speech production) and (ii) they are matched for phonological and semantic content. The values on the y axis (parameter estimates) correspond to those shown in ​Fig. 3 ​for speaking aloud visual words (W) and one-back matching on auditory words (W).

We found a highly significant main effect of hemisphere (F (11.4) p  =  0.003), a 2 way interaction between hemisphere and task (F (19.9) p  =  0.000) and a 3 way interaction between hemisphere, task and region (F (8.7) p  =  0.007). The main effect of hemisphere reflected greater activation in the right than left hemisphere. The 2-way interaction (between (i) hemisphere and (ii) task) arose because activation was higher in the right than left hemisphere during speech production compared to one-back matching. The 3-way interaction (between (i) task, (ii) hemisphere and (iii) region) arose because, for speaking but not one-back matching, there was a greater effect of hemisphere (right  >  left) in pSTS than STG (see Fig. 4). This effect was observed across all speech production conditions (compare first and second rows of Fig. 3), therefore it did not additionally interact with: (i) modality (visual vs auditory), (ii) semantic content (words and pictures > pseudowords and baselines), and/or (iii) phonological content (words and pseudowords  >  pictures and baselines). From these results, we can conclude that the enhanced activation we observed for speech production tasks in pSTS was right lateralised.

#### 3.2.5 Are RpSTS and bilateral STG sensitive to the semantic, phonological or acoustic properties of the auditory input?

In RpSTS, activation was higher for stimuli that lacked semantic content (i.e. pseudowords and baseline conditions) (contrast d2 in Table 1, Table 3) and this was greater in the auditory than visual condition (see contrast (d2 x a) in Table 3). There was no significant interaction between semantic and phonological content or semantic content and task (p  >  0.001 uncorrected). However, there was a weak effect of orthography (contrast f in Table 1, Table 3) because RpSTS was activated by written words and pseudowords more than visual object naming or colour naming. As discussed in the next section, this is attributed to phonological processing of orthographic inputs.

In left and right STG, activation was insensitive to the presence or absence of semantic content (contrasts d and d2 in Table 1) but there was a significant phonology by stimulus modality interaction (contrast (e2 x a) in Table 3) that arose from higher STG activation for auditory stimuli without phonology (sounds and humming). As noted in Section 2.3, these non-phonological auditory conditions had longer stimulus durations than those that did involve phonology. This suggests that our bilateral STG regions of interest were sensitive to the amount of auditory input.

#### 3.2.6 Do RpSTS or bilateral STG also respond in the absence of auditory input?

RpSTS activation was significantly activated during one-back matching on visual pseudowords (at (+45 -30 0) Z score = 4.2). In contrast, neither left nor right STG responded during any of the visual one-back matching conditions.

#### 3.2.7 Summary of results for semantic, phonological and orthographical analyses

To summarise, bilateral STG activation was most sensitive to the demands on nonverbal acoustic processing because, irrespective of task, it was highest for auditory object sounds and vocal humming and lowest for speech stimuli (auditory words and pseudowords). In contrast, RpSTS activation was most responsive when auditory stimuli were devoid of meaning (pseudowords and vocal humming more than words and object sounds) and during phonological processing of orthographic stimuli. RpSTS (but not bilateral STG) also responded in the absence of auditory input (during one-back matching on visually presented pseudowords).

## 4 Discussion

In this study, we found that an auditory processing region in the right posterior superior temporal sulcus (RpSTS) was more responsive during speech production than when listening to auditory stimuli. Enhanced RpSTS activation during speech production was observed (i) in the absence of auditory amplification of the spoken response, (ii) irrespective of whether the stimuli were presented in the visual modality (e.g. reading written words) or auditory modality (e.g. auditory word repetition) and (iii) after controlling for the semantic and phonological content of the heard stimuli (see Fig. 3).

Two previous studies (Christoffels et al., 2007; Agnew et al., 2013) have also reported data indicating that RpSTS activation is higher during speech production than listening. However, the focus of both these studies was to explain how activity in auditory regions was suppressed during speaking compared to listening and neither study expected, statistically tested or interpreted their data showing the reversed effect (i.e. more activation for speaking than listening). Our study is therefore the first to confirm and highlight a special role for RpSTS in speech production. In addition, we investigated the functional properties of RpSTS for the first time, by testing how activation varied over 16 conditions that systematically manipulated the presence or absence of auditory input, semantic content, sublexical phonological cues to speech production and orthographic processing. Our novel findings and conclusions are discussed below.

### 4.1 RpSTS activation was strongly driven by bottom-up auditory input

By definition, RpSTS activation for speaking compared to listening was observed in regions showing a significant main effect of auditory compared to visual stimuli. In addition, we found that RpSTS activation during auditory conditions was significantly higher for unfamiliar than familiar auditory stimuli (i.e. in the absence of semantic content). This unfamiliarity effect for auditory stimuli was observed irrespective of task (i.e. while participants were producing their own speech or listening to auditory input through headphones during one back matching) and was not observed for the visual conditions, irrespective of task (see Fig. 3). Kriegstein and Giraud (2004) have also reported increased RpSTS activation for unfamiliar compared to familiar auditory stimuli.

Plausibly, participants need to attend more closely to the spectral-temporal content of auditory stimuli when semantic cues are not available. Enhanced RpSTS activation during speaking compared to listening might therefore be a consequence of participants attending to the spectral-temporal content of their own speech more when speech was masked by scanner noise rather than heard through earphones. However, this does not explain why Christoffels et al. (2007) and Agnew et al. (2013) also observed increased RpSTS activation for speech production compared to listening when the speech production conditions presented recordings, via earphones, of the participants own speech for the same items.

### 4.2 RpSTS activation responds to phonological stimuli in the absence of auditory inputs

Although RpSTS was strongly driven by auditory inputs, it also responded during one-back matching of written pseudowords in the absence of auditory inputs. To understand how RpSTS contributes to one-back matching of visual pseudowords, we consider the processing stages that may be involved in this task. These are: (i) visual processing; (ii) orthographic processing of letter strings, (iii) links from orthography to phonology (spelling to sound conversion), (iv) short term memory of the visual features, (v) short term memory of orthographic features and (vi) short term memory of phonology features, (vii) comparison of the memory of the stimulus to the next stimulus, (viii) an identity decision (same or different) and (ix) a finger press response. We can rule out RpSTS activation arising at stages: (i), (iv), (vii), (viii) and (ix) because these processes are heavily involved in one-back matching of objects and colours – which did not result in RpSTS activation. Stages (ii) and (iii) are also unlikely to explain RpSTS activation because RpSTS responses were not sensitive to orthographic to phonological processing when reading aloud written words and pseudowords was compared to object and colour naming (see Fig. 3).

On the basis of current evidence, we therefore propose that RpSTS activation during one back matching of visually presented pseudowords is best explained by the demands on short term memory of phonological features following phonological processing of orthographic stimuli. This is consistent with a functional imaging study (Fujimaki et al., 2004) that reported RpSTS activation when participants covertly rehearsed phonological, meaningless sequences of Japanese speech sounds from memory. However, we are not claiming that RpSTS activation is specific to verbal, speech or voice processing. It may also be involved in non-verbal auditory memory. Indeed, right but not left temporal lobectomy was found to impair the ability to retain non-verbal auditory information over short time spans (Zatorre and Samson, 1991).

### 4.3 A role for RpSTS in integrating auditory expectations with spectral-temporal processing of auditory input

We have shown that RpSTS responded independently to both spectral temporal processing of auditory inputs and short term memory of speech sounds consistent with conclusions from Tian and Poeppel (2010) who showed that similar auditory cortical fields mediate both overt auditory perception and auditory imagery. These authors also showed that auditory cortex was more activated for articulation imagery compared with hearing imagery (in the absence of external stimuli, articulatory movement or overt feedback) with this effect located to the right posterior STS (at +54 -26 ​+2) and left anterior STG (at -54 ​+ ​6 -6) (Tian et al., 2016). A third study by Tian and Poeppel (2013) also illustrated how auditory imagery interacts with the effect of auditory input by demonstrating a reduction in RpSTS response to auditory stimuli when participants imagine hearing a cued syllable. These findings also align with those from Wiegand et al. (2018) who found right lateralised responses in RpSTS for conditions that enhanced auditory conscious perception.

Further to these prior findings, we demonstrate that RpSTS is activated during speech production in the absence of experimentally perturbed feedback or articulatory or auditory imagery strategies. Coupled with the results of Tian and Poeppel described above, we propose that RpSTS plays a special role in detecting whether auditory inputs during speech production correspond to higher level expectations of what self-generated speech should sound like. RpSTS may therefore serve to ensure that the sounds produced correspond to the sounds intended and to guide the production of subsequent speech (Levelt, 1983; Tourville et al., 2008; Tourville and Guenther, 2011). This process may involve greater attention to auditory processing in RpSTS during speech production than listening tasks.

We also note that, RpSTS is just one of the many regions where activation increases when auditory feedback during speech production is experimentally perturbed to create a mismatch between what was intended and what was perceived (Behroozmand et al., 2015; Houde and Jordan, 1998; Niziolek and Guenther, 2013; Tourville et al., 2008; Zheng et al., 2010). It is therefore possible that activation in other regions when auditory feedback is experimentally perturbed might reflect acoustic processing or attention that is not typical of normal speech production.

### 4.4 Sensitivity to auditory inputs in bilateral STG

In contrast to RpSTS, bilateral STG regions were not sensitive to the familiarity of the stimulus, instead they showed sensitivity to the duration of auditory input. We found that first, bilateral STG activation was higher for the main effect of auditory input than the main effect of speech production. Second, activation in bilateral STG increased for the non-phonological auditory stimuli (object sounds and humming) that had longer durations than speech sounds (Table 2a and Fig. 3). Third, bilateral STG were not activated in the absence of auditory inputs.

Lower activation for speech production than listening to recordings of another’s voice was observed even when the same words and pseudowords were heard in both conditions. This might be explained by the fact that recordings of another’s speech were presented via earphones whereas own speech was not fed back by earphones (i.e. the acoustic quality was not controlled). However, this does not explain why Christoffels et al. (2007) and Agnew et al. (2013) also observed less STG activation for speech production compared to listening when the speech production conditions presented recordings of the participants own speech via earphones. These studies therefore concluded that they were observing suppression of auditory processing during speech production. We add to this result by showing that bilateral STG are more responsive to the length of the auditory stimuli than to their semantic or phonological content. They are therefore likely to be involved in early auditory processing that is not specific to speech.

### 4.5 Limitations

Previous studies of auditory feedback during speech production have kept the speech production task constant while experimentally manipulating the auditory feedback using frequency shifts (Houde and Jordan, 1998; Niziolek and Guenther, 2013; Tourville et al., 2008), syllable pitch changes (Behroozmand et al., 2015) or background noise (Zheng et al., 2010). As experimental perturbation of auditory feedback introduces acoustic differences and attention demands that are not typical of normal speech conditions, our goal was to measure auditory feedback that was not experimentally altered. We therefore focused on comparing own speech during speech production to another’s speech during listening tasks that did not involve speech production. However, this introduces confounds because the own and another’s speech conditions differ in terms of (i) the task (speech production versus listening) (ii) the acoustic quality of the voices (e.g. pitch, intonation, volume, gender, accent, timber, duration, intensity, temporal dynamics, familiarity), and (iii) the sense of agency.

To overcome differences in acoustic quality and agency, Christoffels et al. (2007) compared own speech during speech production to hearing recordings of own speech during listening. This identified regions of interest (bilateral STG and RpSTS) that were used in the current study. Therefore although our own study cannot exclude the influence of voice and agency differences, these confounds cannot explain why the same regions were associated with own speech processing when acoustic quality and agency were controlled by Christoffells et al. (2007).

To overcome task differences, we focused our analysis on auditory processing regions that were identified as being more activated by all conditions with auditory stimuli compared to all corresponding conditions with visual stimuli (matched for task, semantic content and sublexical phonological cues). Activation in these regions during speech production was therefore primarily driven by auditory processing of the spoken response (i.e. auditory feedback) but we also demonstrate a potential role for RpSTS in phonological short term memory in the absence of auditory input. Other studies are therefore required to investigate the range of processing that involves RpSTS.

Finally, we note that although we did not experimentally manipulate auditory feedback during speech production, the auditory signal would have been affected by the noise of the scanner, particularly since the spoken output was not delivered via earphones. Under these circumstances we would expect auditory feedback to be reduced relative to speaking in a quieter environment or hearing speech during one-back matching. It is therefore surprising that RpSTS activation was higher for speaking than hearing another’s speech via earphones. The enhanced RpSTS activity suggests that participants were actively attending to the spectral temporal features of the auditory feedback in the noisy environment even though the speech production tasks were highly familiar and easy to perform. RpSTS activation in other studies of object naming, reading aloud and auditory repetition is therefore also likely to reflect attention to auditory feedback during speech production.

## 5 Conclusion

Our study has investigated and interpreted a right lateralised response in pSTS during speech production. Activation in this RpSTS region was significantly higher for (i) all auditory compared to all visual stimuli matched for semantic and phonological content, (ii) speech production compared to listening to auditory stimuli during a one-back matching task and (iii) one back matching on written pseudowords in the absence of any auditory input. Based on these and prior findings, we have proposed that the right pSTS region may play a special role in matching auditory expectations with spectral-temporal processing from auditory feedback during speech production.

Our findings complement those that have used experimentally perturbed auditory feedback by highlighting a special role for RpSTS (among the other regions associated with experimentally perturbed speech) and demonstrating that RpSTS is involved in internal representations of speech (i.e. phonology) in addition to bottom up auditory feedback.

Further studies are now needed to understand RpSTS responses further. For example, is the response in RpSTS during speech production proportional to the degree of mismatch between bottom-up inputs and top-down expectations? This could be measured by silencing part of the spoken response fed back to the participant whilst reading aloud pseudowords. The causal relevance of RpSTS to speech production can also be tested by determining whether damage to the RpSTS region we have identified here impairs speech production and/or alters the neural networks that support speech production. It will also be important to understand how RpSTS interacts with other regions if we are to get a full understanding of the neural mechanisms supporting speech production during speech acquisition, adult life, hearing loss and after brain injury.

## Conflicts of Interest

The authors declare no competing financial interests.
